# The Latin American Spanish version of the Face-Name Associative Memory Exam is sensitive to cognitive and pathological changes in preclinical autosomal dominant Alzheimer’s disease

**DOI:** 10.1186/s13195-020-00671-w

**Published:** 2020-09-10

**Authors:** Clara Vila-Castelar, Nathalia Muñoz, Kathryn V. Papp, Rebecca E. Amariglio, Ana Baena, Edmarie Guzmán-Vélez, Yamile Bocanegra, Justin S. Sanchez, Eric M. Reiman, Keith A. Johnson, Reisa A. Sperling, Francisco Lopera, Dorene M. Rentz, Yakeel T. Quiroz

**Affiliations:** 1 Department of Psychiatry, Massachusetts General Hospital, Harvard Medical School, Boston, MA USA; 2 Department of Neurology, Brigham and Women’s Hospital, Harvard Medical School, Boston, MA USA; 3 Department of Neurology, Massachusetts General Hospital, Harvard Medical School, Boston, MA USA; 4 Grupo de Neurociencias de Antioquia, Universidad de Antioquia, Medellín, Colombia; 5 Banner Alzheimer’s Institute, Phoenix, AZ USA; 6 Department of Radiology, Massachusetts General Hospital, Harvard Medical School, Boston, MA USA; 7 Athinoula A. Martinos Center for Biomedical Imaging, Massachusetts General Hospital, Harvard Medical School, Charlestown, MA USA

**Keywords:** Alzheimer’s disease, Autosomal dominant Alzheimer’s disease, Neuropsychology, Associative memory, Imaging, PET

## Abstract

**Background:**

To determine whether performance on the Latin American Spanish version of the Face-Name Associative Memory Exam (LAS-FNAME) can differentiate between cognitively intact carriers of an autosomal dominant Alzheimer’s disease mutation (E280A) in Presenilin-1, who are genetically determined to develop early-onset dementia, from matched non-carriers. We also sought to examine whether LAS-FNAME performance is associated with amyloid-β and regional tau burden in mutation carriers.

**Methods:**

A total of 35 cognitively intact mutation carriers (age range 26–41), 19 symptomatic carriers, and 48 matched non-carriers (age range 27–44) completed a neuropsychological assessment including the LAS-FNAME. A subset of participants (31 carriers [12 symptomatic] and 35 non-carriers) traveled from Colombia to Boston to undergo positron emission tomography (PET) using Pittsburgh compound B to measure mean cortical amyloid-β and flortaucipir for regional tau. ANOVA analyses and Spearman correlations were used to examine group differences and relationships among LAS-FNAME performance and amyloid-β and tau accumulation.

**Results:**

Compared to non-carriers, cognitively intact mutation carriers had lower scores on the LAS-FNAME Total Scores (*p* = .040). Across all carriers (including symptomatic carriers), higher levels of amyloid-β (*r* = − .436, *p* = .018) and regional tau in the entorhinal (*r* = − .394, *p* = .031) and inferior temporal cortex (*r* = − .563, *p* = .001) were associated with lower LAS-FNAME Total Scores.

**Conclusions:**

Performance on the LAS-FNAME differentiated between cognitively intact mutation carriers from non-carriers and was associated with greater amyloid and tau burden when examining all carriers. Findings suggest that the LAS-FNAME is sensitive to early clinical and pathological changes and can potentially help track disease progression in Spanish-speaking individuals.

## Introduction

Alzheimer’s disease (AD) affects 6 million individuals in the USA [1]. Latinos have a higher rate of AD than non-Hispanic whites [2], which is expected to rise rapidly due to population aging [3]. Yet, there is an underrepresentation of Latinos in clinical trials to prevent AD. There are growing efforts to promote recruitment of under-represented groups into research studies. Therefore, there is a need to develop neuropsychological measures to detect AD-pathology in Spanish-speaking individuals and track cognitive changes along the AD-trajectory [4].

We validated the Latin American Spanish-Face-Name Associative Memory Exam (LAS-FNAME) [5], an adaptation of the Face-Name Associative Memory Exam (FNAME) to assess Spanish-speaking individuals. The FNAME is a highly demanding, associative memory test, which tracks memory changes along the AD-spectrum [6–8] that has been shown to be correlated with Aβ accumulation in clinically normal older adults [7, 9] and adults with subjective cognitive decline (SCD) [10]. Recent evidence also showed that performance on a computerized version of the FNAME was associated with cerebrospinal fluid levels of amyloid, phosphorylated tau, and total tau in clinically normal and mild cognitive impairment (MCI) [11]. Importantly, the FNAME engages memory network regions, including the hippocampus, and default mode network regions that are affected early in the disease process [12]. Thus, the FNAME is a challenging and multimodal tool that detects early associative memory changes related to AD.

We leveraged data from the largest kindred with autosomal dominant AD (ADAD) due to a mutation (E280A) in *Presenilin-1* (*PSEN1*), who are virtually destined to develop early-onset AD. We examined whether performance on the LAS-FNAME can distinguish cognitively intact individuals at genetic risk to develop dementia later in life, and whether LAS-FNAME performance is related to Aβ and tau accumulation in the brain. We hypothesized that cognitively intact *PSEN1* mutation carriers would perform worse on the LAS-FNAME than non-carriers and that greater levels of Aβ and tau accumulation would be associated with worse LAS-FNAME performance. We also explored performance on subscales of the LAS-FNAME and its association to AD-related pathology in *PSEN1* mutation carriers.

## Methods

### Study design and participants

One hundred and thirteen individuals from the Colombia-Boston biomarker study (COLBOS) were included. Participants lived in or near Medellín, Colombia, and were recruited from the Alzheimer’s Prevention Initiative registry. Participants were under the age of 50 and had a parent with the autosomal dominant AD due to E280A mutation in *PSEN1*. The clinical staging of this kindred is characterized by an onset of MCI [13] at a median age of 44 years old (95% C.I. = 43, 45 years) and dementia at 49 years old (95% C.I. = 49, 50 years) [14].

Cognitively intact participants had to show no cognitive impairment on a standard cognitive battery, including a Mini-Mental State Examination (MMSE) [15] score ≥ 26 and a Functional Assessment Staging test (FAST) [16] = 1 (indicating no cognitive impairment or subjective cognitive concerns). Thirty-five cognitively intact participants were *PSEN1* mutation carriers (mean age 32.14 ± 3.73 years; 51% females), and forty-eight were age-matched non-carrier family members (mean age 34.43 ± 4.32 years; 56% females) (Table 1). Symptomatic carriers (*n* = 19) were defined as having a FAST score of 2, indicating the presence of subjective cognitive concerns (*n* = 11, mean age 38.36 ± 4.92), or a FAST score of 3, indicating the presence of MCI (*n* = 8, mean age 43.75 ± 2.91). Mutation carriers and non-carriers were age-matched. Potential participants were screened and excluded in advance for the presence of neurological or chronic psychiatric disorders, and eligibility to undergo MRI. Participants and investigators were blind to genetic status.

**Table 1:** Demographic, cognitive, and clinical characteristics of the sample

	Non-carriers (***n =*** 48)	Cognitively intact carriers (***n =*** 35)	*p*^a^	Symptomatic carriers (***n*** = 19)	*p*^b^
	M (SD)	M (SD)		M (SD)	
**Demographic and clinical variables**					
Age (years)	34.43 (4.32)	32.14 (3.73)	.018*	40.63 (4.92)	.557
Education (years)	10.45 (4.19)	10.72 (3.68)	.769	7.16 (3.84)	.259
MMSE	28.94 (.88)	28.67 (.93)	.199	25.63 (3.17)	.001*
GDS	1.00 (.00)	1.03 (.00)	1.000	2.84 (3.35)	.798
**Cognitive variables**					
CERAD Word List Learning	20.63 (2.98)	20.28 (3.54)	.799	13.84 (4.39)	.007*
CERAD Word List Delayed Recall	7.67 (1.26)	7.31 (1.58)	.534	3.74 (2.64)	.002*
CERAD Word List Recognition	9.71 (.58)	9.81 (.47)	.486	7.95 (2.20)	.034*
CERAD Constructional Praxis-Copy	10.41 (.89)	10.25 (.91)	.346	9.53 (1.12)	.030*
CERAD Constructional Praxis-Delayed Recall	9.57 (1.70)	9.72 (1.52)	.744	4.63 (3.48)	.036*
Phonemic Fluency (FAS)	32.41 (7.33)	34.53 (7.89)	.212	31.47 (10.38)	.868
Semantic Fluency (Animals)	20.37 (4.01)	21.31 (4.20)	.233	18.74 (3.97)	.888
Boston Naming Test (BNT-15)	13.82 (.95)	13.33 (1.24)	.055	12.95 (1.71)	.023*
**PET variables^**					
Amyloid (Aβ) (DVR)	1.11 (0.04)	1.45 (.24)	.000*	1.93 (.30)	.000*
Entorhinal cortex tau (SUVR)	1.05 (0.21)	1.34 (.42)	.054	2.10 (.70)	.000*
Inferior temporal cortex tau (SUVR)	1.18 (0.11)	1.23 (.16)	.108	1.72 (.46)	.001*

*Note:* Symptomatic carriers include participants with FAST scores of 2 and 3 indicating presence of SCD and MCI, respectively

*Abbreviations*: *M* mean, *SD* standard deviation, *FAST* Functional Assessment Staging test, *MMSE* Mini Mental State Exam, *GDS* Geriatric Depression Scale, *CERAD* Consortium to Establish a Registry for Alzheimer’s Disease, *DVR* distribution volume ratio, *SUVR* standardized uptake value ratio

^From subset participants with PET data. All PET values were partial volume corrected (PVC)

^a^*p* value calculated for Mann-Whitney test for cognitively intact *PSEN1* mutation carriers versus non-carriers

^b^*p* value calculated for Mann-Whitney test for all *PSEN1* mutation carriers (i.e., cognitively intact, SCD, and MCI) versus non-carriers

*p < 0.05. Group differences significant at the 0.05 level (2-tailed)

### Procedures

Ethics approval was obtained from the University of Antioquia (Colombia) Ethics Committee and the Partners Human Research Institutional Review Board. Clinical and cognitive evaluations (including the LAS-FNAME task) were administered in Spanish by psychometrists and neuropsychologists at the Grupo de Neurociencias de Antioquia within 2 months of brain imaging. One hundred and two individuals completed these evaluations, including 35 cognitively intact mutation carriers (age range 26–41), 48 matched non-carriers (aged 27–44), and 19 symptomatic carriers (13 SCD and 6 MCI). A subset of participants (*n* = 66), 31 carriers (6 SCD, 6 MCI) and 35 non-carriers, traveled to Boston to complete magnetic resonance imaging (MRI) and positron emission tomography (PET) imaging. One participant could not complete the 11C Pittsburgh compound B (PiB) PET scan due to radiotracer production failure.

Cognitive testing included the Consortium to Establish a Registry for Alzheimer’s Disease neuropsychological battery (CERAD) word list and constructional praxis, Boston Naming Test (BNT-15), MMSE, Geriatric Depression Scale [17] and FAST [16].

### LAS-FNAME task description

Detailed description of this task can be found elsewhere [5]. In brief, during the initial learning, participants are shown a succession of 12 faces with a name written below on a computer screen. Participants are asked to recognize the target face between two distractors (Immediate Recognition—Face), provide the initial letter of the name associated with the learned face (Immediate Recall—Letter), and finally, identify the name that was paired with each face among three choices (Immediate Recognition—Name). Items correctly identified for each condition (Face, Letter, Name) are summed for an Immediate Memory Total score (/36).

Following a 25-min delay, participants are asked to identify again the learned face between two distractors (Delayed Recognition—Face), recall the initial letter of the name associated with each face (Delayed Recall—Letter), and select the name per face among three choices (Delayed Recognition—Name). Items correctly identified for each condition (Face, Letter, Name) are summed for a Delayed Memory Total score (/36). Immediate and Delayed Memory total scores are summed to produce a LAS-FNAME Total Score (/72).

### Brain imaging

Participants underwent tau and amyloid PET imaging at Massachusetts General Hospital, Boston. As previously reported [18], PiB PET was acquired with a 8.5–15 mCi bolus injection followed immediately by a 60-min dynamic acquisition in 69 frames (12 × 15 s, 57 × 60 s). [F18] Flortaucipir (FTP) was acquired between 80 and 100 min after a 9.0–11.0 mCi bolus injection in 4 separate 5-min frames.

11C PiB PET data were expressed as the distribution volume ratio (DVR) with cerebellar gray matter as reference tissue; regional time-activity curves were used to compute regional DVRs for each region of interest (ROI) using the Logan graphical method applied to data obtained between 40 and 60 min after injection [19]. 11C PiB retention was assessed using a large cortical ROI aggregate that included frontal, lateral temporal, and retrosplenial cortices [20].

[F18] FTP-specific binding was expressed in FreeSurfer ROIs as the standardized uptake value ratio (SUVR) to cerebellar gray matter [21]. We selected FTP binding on bilateral entorhinal and inferior temporal cortices (EC and IT) as these regions are both crucial for associative memory, and early sites of tau accumulation in ADAD [18]. PET data were corrected for partial volume effects using the geometric transfer matrix method [22]. For whole-brain analyses of PiB and FTP, DVR and SUVR images were normalized to standard space and smoothed with an 8-mm Gaussian kernel to account for individual anatomic differences [23].

### Statistical analyses

Statistical analyses were performed using SPSS [24]. Mann-Whitney tests were conducted to compare demographic and clinical characteristics between *PSEN1* mutation carriers and non-carriers. An analysis of variance was used to compare LAS-FNAME Total Score, between cognitively intact *PSEN1* mutation carriers and non-carriers. We then conducted the same analysis including all *PSEN1* mutation carriers (i.e., cognitively intact, SCD, and MCI) and matched non-carriers to examine whether the LAS-FNAME was sensitive to capture cognitive changes along the disease trajectory. Effect sizes (i.e., Cohen’s *d*) and models adjusting for age and years of education are reported. Spearman’s correlations examined the relationship between LAS-FNAME Total Score, mean cortical Aβ and regional tau accumulation in the EC and IT in cognitively intact *PSEN1* mutation carriers to examine how performance on the LAS-FNAME was associated with early markers of AD-pathology accumulation. We then conducted the same analysis across all *PSEN1* mutation carriers to examine how performance on the LAS-FNAME relates to accumulation of pathology through preclinical stages. No correction for multiple comparisons was employed due to small sample size.

Exploratory analyses compared performance on the LAS-FNAME subscales (i.e., Immediate Memory Total and Delayed Memory Total) and conditions (i.e., Face Recognition, Letter Recall, and Name Recognition) between *PSEN1* mutation carriers and non-carriers. Lastly, we carried out exploratory whole-brain analyses examining the relationship between Aβ and tau burden and LAS-FNAME Total Score in *PSEN1* mutation carriers. Regions were *p* < 0.05 after cluster-wise false discovery rate correction for multiple comparisons (minimum cluster extent *k* = 100 mm^2^).

## Results

### Demographic, clinical, and cognitive measures in carriers and non-carriers

Demographic, cognitive, and clinical data are presented in Tables 1 and 2. Cognitively intact *PSEN1* mutation carriers were significantly younger than non-carriers (*p* = .018). There were no differences in educational level or percentage of females. Cognitively intact *PSEN1* mutation carriers had slightly lower BNT-15 scores (*p* = .055) compared to non-carriers. No other differences were found among cognitive measures. As expected, symptomatic carriers were older, had lower MMSE scores, and performed worse across most cognitive measures than non-carriers.

**Table 2:** Demographic and clinical characteristics of subset participants with PET data

	Non-carriers (***n*** = 35)	Cognitively intact carriers (***n*** = 19)	Symptomatic carriers (***n*** = 12)
	*M (SD)*		
**Demographic and clinical variables**			
Age (years)	34.69 (4.84)	33.95 (5.08)	42.25 (3.41)
Education (years)	11.03 (4.16)	11.21 (3.15)	5.75 (3.14)
MMSE	28.97 (.92)	28.68 (.88)	25.08 (3.23)
GDS	.94 (1.49)	.63 (1.46)	2.75 (3.22)
**Cognitive variables**			
CERAD Word List Learning	20.91 (3.15)	20.05 (2.99)	12.50 (3.87)
CERAD Word List Delayed Recall	7.69 (1.25)	7.05 (1.96)	2.92 (2.39)
CERAD Word List Recognition	9.74 (.56)	9.84 (.37)	7.33 (2.27)
CERAD CP- Copy	10.40 (1.03)	10.58 (.61)	9.50 (1.31)
CERAD CP- Delayed Recall	9.51 (1.58)	10.05 (1.58)	3.83 (3.43)
Phonemic Fluency (FAS)	32.54 (7.69)	36.74 (7.96)	29.17 (8.40)
Semantic Fluency (Animals)	20.00 (3.66)	21.68 (4.70)	18.25 (3.93)
Boston Naming Test (BNT-15)	13.86 (1.00)	13.58 (1.17)	13.00 (1.76)

*Note:* Symptomatic carriers include participants with FAST scores of 2 and 3 indicating presence of SCD concerns and MCI, respectively

*Abbreviations: M* mean, *SD* standard deviation, *FAST* Functional Assessment Staging test, *MMSE* Mini Mental State Exam, *GDS* Geriatric Depression Scale, *CERAD* Consortium to Establish a Registry for Alzheimer’s Disease, *CP* constructional praxis

### LAS-FNAME performance in *PSEN1* mutation carriers and non-carriers

**Fig. 1:**
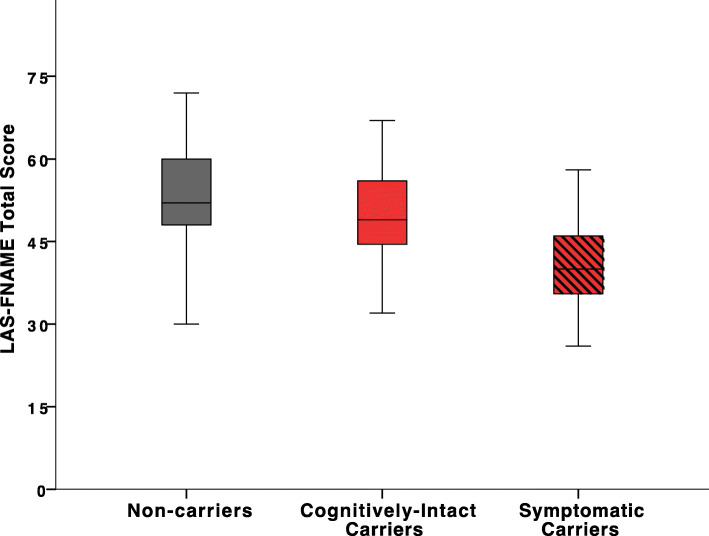
Note. Performance on the LAS-FNAME Total Score in *PSEN1* mutation carriers and non-carriers. Cognitively intact *PSEN1* mutations carriers had significantly lower LAS-FNAME Total Score (*p = .*040) than non-carriers. Across all *PSEN1* mutation carriers (i.e., cognitively intact, subjective cognitive decline, and mild cognitive impairment), carriers had significantly lower LAS-FNAME Total Score (*p* = .001) than non-carriers

Cognitively intact *PSEN1* mutations carriers had significantly lower scores on the LAS-FNAME Total Score (*p = .*040) and Immediate Memory (*p = .*037) and showed a trend towards lower scores on Delayed Memory (*p* = .059) than non-carriers (Table 3 and Fig. 1). When adjusting for age and years of education, cognitively intact carriers showed a trend towards lower scores on the Immediate Memory (*F* (1, 83) = 3.92, *p* = .051), Delayed Memory (*F* (1, 83) = 2.92, *p* = .092), and LAS-FNAME Total Score (*F* (1, 83) = 3.64, *p* = .060).

**Table 3:** Differences in LAS-FNAME performance between cognitively intact *PSEN1* mutation carriers and non-carriers

	Non-carriers (***n =*** 48)	Cognitively intact carriers (***n =*** 35)				
LAS-FNAME	*M (SD)*	*M (SD)*	*df*	*F*	*p* value^a^	*d*
**Immediate Memory**						
Face Recognition	11.71 (.79)	11.60 (.74)	1, 83	.40	.530	.14
Letter Recall	4.88 (2.60)	4.20 (2.29)	1, 83	1.50	.225	.28
Name Recognition	9.83 (1.70)	8.54 (2.45)	1, 83	8.09	.006*	.62
**Delayed Memory**						
Face Recognition	11.81 (.53)	11.80 (2.45)	1, 83	.10	.919	.01
Letter Recall	6.06 (2.98)	4.80 (2.42)	1, 83	4.24	.043*	.47
Name Recognition	9.27 (2.39)	8.46 (2.62)	1, 83	2.17	.145	.32
**Scale Scores**						
IM Total	26.42 (4.19)	24.34 (4.66)	1, 83	4.51	.037*	.47
DM Total	27.14 (4.88)	25.06 (4.93)	1, 83	3.67	.059	.42
LAS-FNAME Total	53.56 (8.76)	49.40 (9.26)	1, 83	4.35	.040*	.46

*Abbreviations: M* mean, *SD* standard deviation, *IM* Immediate Memory, *DM* Delayed Memory, *LAS-FNAME Total* IM Total + DM Total, *df* degrees of freedom, *F F*-ratio, *d* Cohen’s *d* effect size

^a^*p* value calculated for ANOVA tests for cognitively intact *PSEN1* mutation carriers versus non-carriers

*p < 0.05

Exploratory analyses showed that within Immediate Memory conditions, cognitively intact *PSEN1* mutation carriers had significantly lower scores on Name Recognition (*p = .*006) than non-carriers. Within Delayed Memory conditions, cognitively intact *PSEN1* mutation carriers performed significantly lower on Letter Recall (*p = .*043) than non-carriers.

Performance on the LAS-FNAME across all *PSEN1* mutation carriers showed that symptomatic carriers had significantly lower LAS-FNAME Total Score (*p* = .001), Immediate Memory (*p* = .001), and Delayed Memory (*p* = .001) than non-carriers (Table 4). When adjusting for age and years of education across all mutation carriers, carriers exhibited significantly lower Immediate Memory (*F* (1, 111) = 11.83, *p* = .001), Delayed Memory (*F* (1, 111) = 10.67, *p* = .001), and LAS-FNAME Total Score (*F* (1, 111) = 12.13, *p* = .001).

**Table 4:** Differences in LAS-FNAME performance between symptomatic *PSEN1* mutation carriers and non-carriers

	Non-carriers (***n =*** 48)	Symptomatic carriers (***n*** = 19)				
LAS-FNAME	*M (SD)*	*M (SD)*	*df*	*F*	*p* value^a^	*d*
**Immediate Memory**						
Face Recognition	11.71 (.79)	10.84 (1.71)	1, 111	2.53	.114	.38
Letter Recall	4.88 (2.60)	2.84 (1.89)	1, 111	5.25	.024*	.49
Name Recognition	9.83 (1.70)	7.00 (2.21)	1, 111	18.61	.000*	.86
**Delayed Memory**						
Face Recognition	11.81 (.53)	10.58 (1.80)	1, 111	3.68	.058	.47
Letter Recall	6.06 (2.98)	3.11 (2.05)	1, 111	11.12	.001*	.70
Name Recognition	9.27 (2.39)	7.11 (1.91)	1, 111	6.71	.011*	.52
**Scale Scores**						
IM Total	26.42 (4.19)	20.68 (4.40)	1, 111	12.58	.001*	.75
DM Total	27.14 (4.88)	20.79 (4.43)	1, 111	11.45	.001*	.71
LAS-FNAME Total	53.56 (8.76)	41.47 (8.32)	1, 111	12.77	.001*	.76

*Note:* Symptomatic carriers include participants with FAST scores of 2 and 3 indicating presence of subjective cognitive concerns and mild cognitive impairment, respectively

*Abbreviations: M* mean, *SD* standard deviation, *IM* Immediate Memory, *DM* Delayed Memory, *LAS-FNAME Total* IM Total + DM Total, *df* degrees of freedom, *F* F-ratio, *d* Cohen’s d effect size

^a^*p* value calculated for ANOVA tests for all *PSEN1* mutation carriers (i.e., cognitively intact, subjective cognitive concerns, and mild cognitive impairment) versus non-carriers

*p < 0.05

Exploratory analyses showed that within conditions, symptomatic carriers performed significantly lower on Immediate Memory—Letter Recall (*p* = .024) and Name Recognition (*p* = .000), as well as Delayed Memory—Letter Recall (*p* = .001) and Name Recognition (*p* = .011) than non-carriers. Symptomatic *PSEN1* mutation carriers showed a trend towards lower scores on Delayed Memory—Face Recognition (*p* = .058), while Immediate Memory—Face Recognition was not significantly different than in non-carriers (*p* = .114).

### Age and LAS-FNAME performance in *PSEN1* mutation carriers

**Fig. 2:**
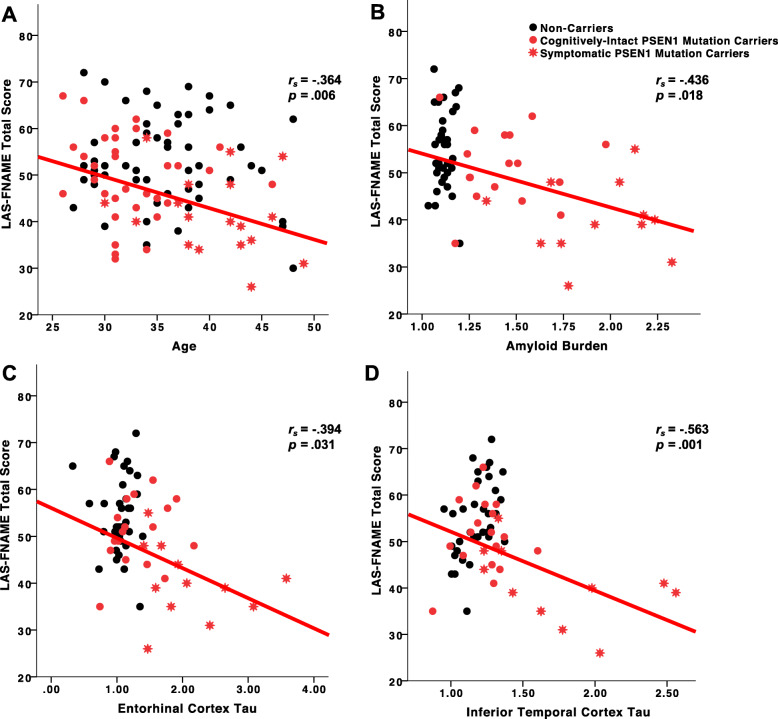
Associations among age, amyloid, regional tau burden, and LAS-FNAME. **a** LAS-FNAME Total Score as a function of Age in *PSEN1* mutation carriers and non-carriers. **b** LAS-FNAME Total Score as a function of amyloid-β accumulation in *PSEN1* mutation carriers and non-carriers. **c** LAS-FNAME Total Score as a function of tau accumulation in the entorhinal cortex in *PSEN1* mutation carriers and non-carriers. **d** LAS-FNAME Total Score as a function of tau accumulation in the inferior temporal cortex in *PSEN1* mutation carriers and non-carriers. Black circles represent non-carriers, red circles represent cognitively intact *PSEN1* mutation carriers, and red stars represent symptomatic *PSEN1* mutation carriers. Spearman’s rho coefficient and *p* values across all PSEN1 mutation carriers are shown. Linear fit line is shown for all *PSEN1* mutation carriers to aid inspection

Age was negatively correlated with performance on the LAS-FNAME, wherein older cognitively intact *PSEN1* mutation carriers have lower LAS-FNAME Total (*r* = − .163; *p* = .350), Immediate Memory (*r* = − .190; *p* = .275), and Delayed Memory score (*r* = − .109; *p* = .531); however, these associations did not reach significance (Fig. 2a). In contrast, when examining the relationship between age and LAS-FNAME scores across all carriers, greater age was significantly associated with lower performance on the LAS-FNAME Total Score (*r* = −.364, *p* = .006) and all its subscales (Immediate Memory: *r* = − .328, *p* = .014; Delayed Memory: *r* = − .356, *p* = .008).

### Aβ burden and LAS-FNAME performance in *PSEN1* mutation carriers

Cognitively intact *PSEN1* mutation carriers had significantly higher Aβ burden than non-carriers (*p* = .000). Aβ burden was significantly higher across all *PSEN1* mutation carriers than in non-carriers (*p* = .000) (Table 1).

There was no association between performance on the LAS-FNAME and Aβ burden in cognitively intact *PSEN1* mutation carriers (Immediate Memory: *r* = − .111, *p* = .683; Delayed Memory: *r* = − .077, *p* = .777; Total Score: *r* = − .016, *p* = .952). We then examined the association between Aβ burden and performance on the LAS-FNAME across all *PSEN1* mutation carriers and found that greater Aβ burden was significantly associated with lower LAS-FNAME Total Score (*r* = − .436, *p* = .018) (Fig. 2b), Immediate Memory (*r* = − .438, *p* = .017), and Delayed Memory (*r* = − .417, *p* = .024) scores.

### Tau and LAS-FNAME performance in *PSEN1* mutation carriers

Cognitively intact *PSEN1* mutation carriers showed a trend towards higher levels of EC tau (*p* = .054) than non-carriers, while there was no difference in IT tau (*p* = .108). Compared to non-carriers, both EC and IT tau were significantly higher across all carriers (EC tau: *p* = .000; IT tau: *p* = .001) (Table 1).

Among cognitively intact *PSEN1* mutation carriers, performance on the LAS-FNAME was not associated with EC tau (Immediate Memory: *r* = − .085, *p* = .746; Delayed Memory: *r* = .303, *p* = .237; Total Score: *r* = .236, *p* = .362). When examining these associations across all *PSEN1* mutation carriers, we found that greater EC tau was associated with lower LAS-FNAME Total Score (*r* = − .394, *p* = .031) (Fig. 2c) and Immediate Memory scores (*r* = − .435, *p* = .016), while Delayed Memory scores showed a trend towards significance (*r* = − .350, *p* = .058).

Finally, we examined the association between IT tau and LAS-FNAME scores and found that among cognitively intact *PSEN1* mutation carriers, LAS-FNAME scores were not associated with IT tau (Immediate Memory: *r* = − .188, *p* = .469; Delayed Memory: *r* = .086, *p* = .742; Total Score: *r* = − .082, *p* = .754). In contrast, across all *PSEN1* mutation carriers, greater IT tau was significantly associated with lower LAS-FNAME Total Score (*r* = − .563, *p* = .001) (Fig. 2d), Immediate Memory Total (*r* = − .570, *p* = .001) and Delayed Memory Total scores (*r* = − .528, *p* = .003).

### Whole-brain analyses of the relationship between pathology burden and LAS-FNAME

**Fig. 3:**
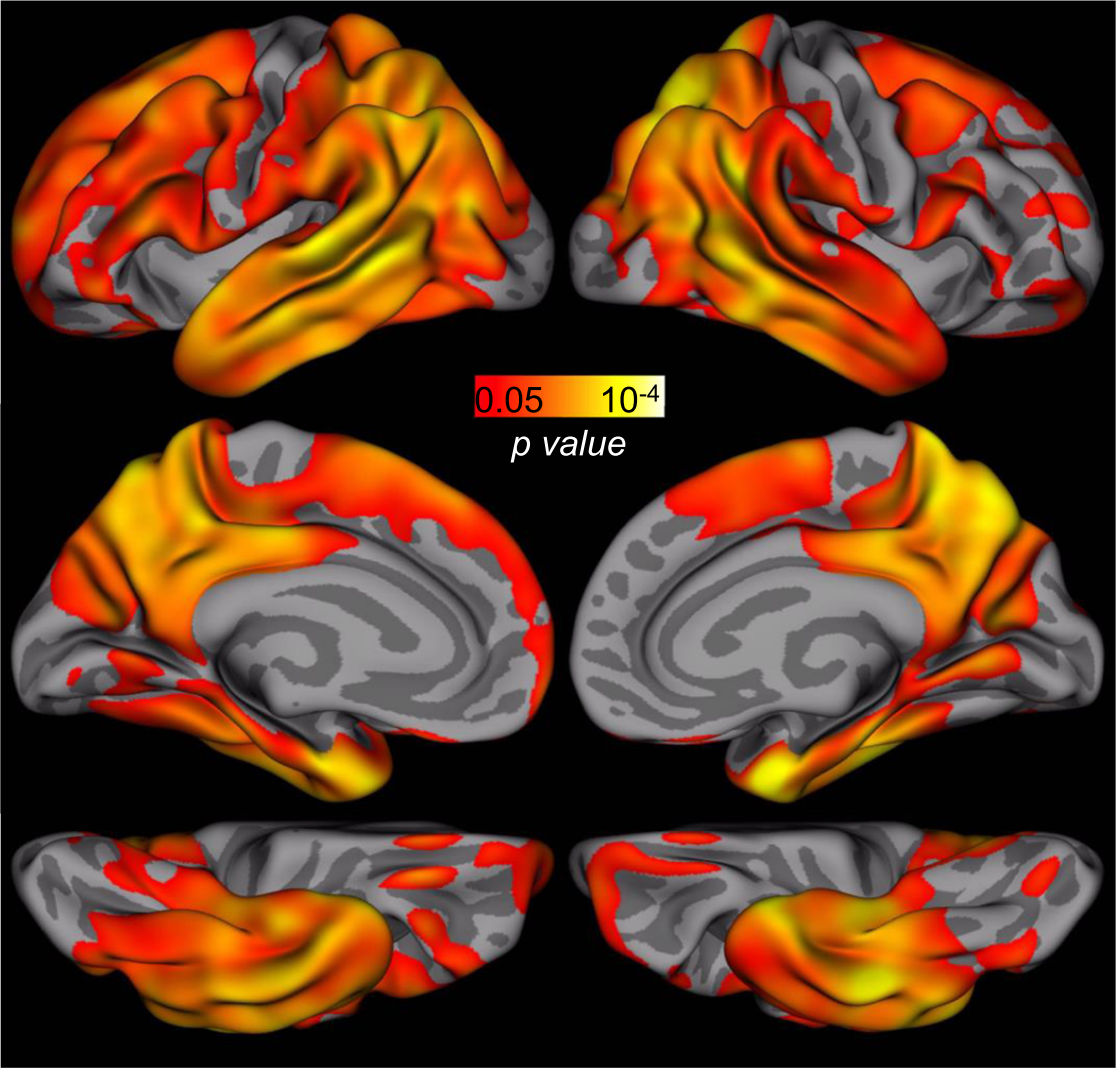
Whole-brain relationships between tau PET measures and LAS-FNAME Total Score. Note. Whole-brain relationships between tau PET measures and LAS-FNAME Total Score within *PSEN1* E280A carriers. Results are displayed as -log_10_(*p*), *p* < 0.05 after cluster-wise false discovery rate (FDR) correction for multiple comparisons, minimum cluster size = 100 mm^2^. No relationships between PiB PET and LAS-FNAME Total Score were significant after FDR correction

We examined the relationship between LAS-FNAME Total Score and Aβ and tau burden in the whole brain within *PSEN1* carriers. Whole-brain analyses showed no significant relationships between Aβ burden and LAS-FNAME Total Score after FDR correction for multiple comparisons. In contrast, whole-brain analyses between tau burden and LAS-FNAME Total Score showed a pattern consistent with findings from regions selected a priori, wherein LAS-FNAME Total Score was related to higher tau burden in inferior and medial temporal regions (i.e., EC and IT), and parietal regions (i.e., precuneus and inferior parietal) (Fig. 3).

## Discussion

The FNAME is an associative memory test sensitive to early changes in AD, which has been associated with Aβ and CSF tau accumulation in clinically normal [7, 9], SCD [10], and MCI [11]. Our aim was to explore whether the LAS-FNAME can help identify individuals at increased risk for developing AD by differentiating between cognitively intact *PSEN1* mutation carriers and non-carriers. Further, we explored the relationship among performance on the LAS-FNAME and in vivo markers of Aβ and tau accumulation in *PSEN1* mutation carriers who underwent PET imaging.

Our findings suggest that the LAS-FNAME can distinguish between cognitively intact *PSEN1* mutation carriers and non-carriers. Cognitively intact carriers had lower LAS-FNAME Total Scores despite being significantly younger than non-carriers and showed a trend towards lower LAS-FNAME Total Scores when adjusting for age and years of education. These findings are remarkable as we defined cognitively intact carriers using stringent criteria, namely absence of objectively defined cognitive impairment and subjective cognitive concerns. Notably, no other cognitive measure was able to distinguish between cognitively intact carriers and non-carriers. Differences in LAS-FNAME scores were even greater when examining across all PSEN1 mutation carriers by including symptomatic carriers (i.e., subjective cognitive decline and mild cognitive impairment) who were further along the disease trajectory. Thus, our findings highlight the potential of the LAS-FNAME as a sensitive cognitive measure to capture subtle memory changes that appear in the early, preclinical stages of AD.

In autosomal dominant AD, age is commonly used as a proxy of disease progression, because as carriers age, they get closer to their age of estimated symptom onset. We found that age and performance on the LAS-FNAME were not significantly associated in cognitively intact *PSEN1* mutation carriers. However, when examining this relationship across all carriers, older age was associated with lower performance on the LAS-FNAME, suggesting that the LAS-FNAME is sensitive to progression of associative memory difficulties from preclinical to mildly symptomatic stages of AD. These findings are also notable in that our sample is remarkably younger than previous studies, highlighting the potential applicability of the FNAME across age ranges.

Our findings suggest that Aβ burden was not associated with LAS-FNAME scores in cognitively intact *PSEN1* mutation carriers whereas higher Aβ burden was associated with lower LAS-FNAME Total Scores when including mildly symptomatic PSEN1 mutation carriers (i.e., carriers with subjective cognitive decline and MCI). Previous research has shown that the FNAME was associated with Aβ burden in clinically normal older adults [7, 9]. However, our findings do not support such association in cognitively intact mutation carriers. This discrepancy is likely due to the sample’s limited age range, which also leads to restricted pathology levels, as *PSEN1* mutation carriers show consistently elevated levels of amyloid at preclinical stages [18]. Thus, when including carriers who are further along the disease and have greater levels of amyloid, the range of Aβ burden values increased, corroborating the association between Aβ burden and LAS-FNAME performance.

To our knowledge, this is the first study investigating the relationship between FNAME performance and PET tau pathology. We examined regional tau accumulation in the entorhinal and inferior temporal cortices, as these regions are both crucial for associative memory [25–27] and sensitive to early disease pathology [18]. We found no association among cognitively intact *PSEN1* mutation carriers. We then examined these associations among cognitively intact and symptomatic PSEN1 mutation carriers and found that greater tau accumulation in the EC and IT were associated with lower LAS-FNAME Total Score. Limited range of tau values among cognitively intact *PSEN1* mutation carriers may explain the lack of association between LAS-FNAME and tau burden. Alternatively, our findings may also suggest that higher levels of tau are required to detect an association between tau burden and LAS-FNAME, as in this kindred, tau pathology is not observed in temporal regions until close to the clinical onset. Notably, when examining these associations in mildly symptomatic individuals further along the AD trajectory, by including carriers with subjective cognitive concerns and mild cognitive impairment, we found that the LAS-FNAME was associated with tau burden in the entorhinal and inferior temporal cortices, early sites of tau accumulation. Moreover, whole-brain analyses confirmed that performance on the LAS-FNAME was related to higher tau burden in inferior and medial temporal regions, including entorhinal and inferior temporal cortices, as well as parietal regions such as the precuneus and inferior parietal. Overall, our findings suggest that the LAS-FNAME is sensitive to early increases in tau pathology before dementia onset.

### Limitations

This study also had some limitations. First, correction for multiple comparisons was not employed due to concerns regarding relatively small sample size and power limitations. Nonetheless, this is a rare and homogeneous sample, as all carriers had a single mutation in P*SEN1* and a well-characterized clinical and disease course. Furthermore, a relatively small sample size among participants who underwent PET imaging may have limited our ability to detect associations between LAS-FNAME performance and pathology burden among cognitively intact *PSEN1* mutation carriers. Lastly, it will be important to investigate how our findings generalize to other AD-causing mutations and the larger sporadic AD population. Future work will require longitudinal data to evaluate whether the LAS-FNAME can track cognitive changes across the AD-spectrum.

## Conclusions

Performance on the LAS-FNAME was able to distinguish cognitively intact *PSEN1* mutation carriers from non-carriers suggesting that the LAS-FNAME can detect early, subtle cognitive changes in individuals who will develop AD with virtual certainty. Furthermore, lower performance on the LAS-FNAME was associated with AD-related pathology accumulation across mildly symptomatic mutation carriers. These findings suggest that subtle changes in associative memory are evident in individuals at genetic risk for AD and are related to AD-related pathology, even early in the disease process, before dementia onset. Thus, the LAS-FNAME may be a useful tool for the detection of preclinical and early AD in Spanish-speaking individuals. Our findings promote efforts to recruit Spanish-speaking individuals from under-represented groups in research studies and ongoing clinical trials to prevent AD.

## Data Availability

Anonymized clinical, genetic, and imaging data are available upon request, subject to an internal review by Y.T.Q. and F.L. to ensure that the participants’ anonymity, confidentiality, and PSEN1 E280A carrier or non-carrier status are protected. Data requests will be considered based on a proposal review, and completion of a data sharing agreement, in accordance with the University of Antioquia and MGH institutional guidelines. Please submit data requests to Y.T.Q. (yquiroz@mgh.harvard.edu)

## Abbreviations

AD: Alzheimer’s disease
LAS-FNAME: Latin American Spanish- Face-Name Associative Memory Exam
FNAME: Face-Name Associative Memory Exam
Aβ: Amyloid-beta
SCD: Subjective cognitive decline
MCI: Mild cognitive impairment
ADAD: Autosomal dominant Alzheimer’s disease
PSEN1: Presenilin-1
MMSE: Mini-Mental State Examination
FAST: Functional Assessment Staging test
MRI: Magnetic resonance imaging
PET: Positron emission tomography
CERAD: Consortium to Establish a Registry for Alzheimer’s Disease
BNT: Boston Naming Test
PiB: 11C Pittsburg Compound B
FTP: [F18] Flortaucipir
DVR: Distribution volume ratio
ROI: Region of interest
SUVR: Standardized uptake value ratio
EC: Entorhinal cortex
IT: Inferior temporal cortex
